# Durability Properties of Macro-Polypropylene Fiber Reinforced Self-Compacting Concrete

**DOI:** 10.3390/ma17020284

**Published:** 2024-01-05

**Authors:** Yaqin Chen, Muhammad Shukat Waheed, Shahid Iqbal, Muhammad Rizwan, Shah Room

**Affiliations:** 1 School of Civil Engineering and Architecture, Xi’an University of Technology, Xi’an 710048, China; chenyaqin2008@126.com; 2 Department of Civil Engineering, Sarhad University of Science and Information Technology, Peshawar 25000, Pakistan; shukatwaheed112@gmail.com (M.S.W.); shahid.civil@suit.edu.pk (S.I.); rizwan.civil@suit.edu.pk (M.R.); 3 Muhandis Design Ingenieure (MDI), Peshawar 25000, Pakistan

**Keywords:** macro-polypropylene, self-compacting concrete, freezing and thawing, acid attack, sulfate attack, fibers, bridging effect

## Abstract

Concrete is one of the most commonly used construction materials; however, its durability plays a pivotal role in areas where the concrete is exposed to severe environmental conditions, which initiate cracks inside and disintegrate it. Randomly distributed short fibers arrest the initiation and propagation of micro-cracks in the concrete and maintain its integrity. Traditional polypropylene fibers are thin and encounter the problem of balling effects during concrete mixing, leading to uneven fiber distribution. Thus, a new polypropylene fiber is developed by gluing thin ones together, forming macro-polypropylene fibers. Thus, different amounts of fibers, 0–1.5% v/f with an increment of 0.5% v/f, are used in different grades of concrete to study their impact on durability properties, including resistance to freezing and thawing cycles, sulfate, and acid attacks. A total of 432 cube samples were tested at 28, 56, and 92 days. The results reveal that the maximum durability, in terms of compressive strength loss, is noted with a fiber content of 1% with improved resistance of 72%, 54%, and 24% against freeze–thaw cycles, sulfate attack, and hydrochloric acid attack, respectively, at 92 days. Thus, the resulting fiber-reinforced concrete may be effective in areas where these extreme exposure conditions are expected.

## 1. Introduction

Concrete is one of the most extensively used construction materials worldwide. However, it has many restrictions in engineering applications due to its poor toughness, high brittleness, low tensile strength and, easy cracking [1,2,3]. Recent research on concrete performance has shown that adding various discontinuous fibers into concrete can significantly improve its natural defects [4,5,6]. Therefore, different types of natural and artificial fibers are added to concrete to investigate their impacts [7,8]. Self-compacting concrete (SCC) is a particular type of concrete that can flow under its own weight to fill the formwork and does not require mechanical vibration for compaction [9], resulting in improvement in the construction site environment by reducing noise, savings in labor cost, and facilitation of the construction process. A higher amount of powder and a high dosage of high-range water-reducing agents are needed to achieve high flow in SCC. Using only cement as the powder content may be uneconomical and lead to micro-cracking in concrete. Mineral fillers, which commonly include blast furnace slag, silica fumes, fly ash, marble powder, and glass powder, are used to solve the problem [10,11,12,13,14]. A previous study has also reported improvements in the resistance of concrete against chemical attack and freezing and thawing by incorporating fly ash into it [15]. Additionally, the maximum aggregate size in the case of SCC is smaller to facilitate ease of flow. The use of more powder content and smaller aggregate size may result in a finer microstructure, which benefits its bonding with fibers. Additionally, the use of mechanical vibration to compact concrete containing distributed short fibers may disturb their natural orientation, resulting in uneven fiber distribution; thus, the use of SCC when different types of fibers are added is beneficial and desirable.

Different short fibers are added to concrete to improve its short- and long-term performance. They are natural as well as artificial fibers. The use of natural fibers in concrete improves its mechanical properties [8]. Similarly, the use of natural fibers in SCC up to a certain limit enhances the mechanical properties and curtails micro-cracks and ductile performance [16]; however, the use of natural fibers can affect the durability characteristics of SCC [17] because they tend to decompose over time. Other problems associated with these fibers are their lower strength compared to artificial fibers and their significant water absorption, which may hinder the hydration of cement in concrete and give rise to workability issues. Thus, artificial fibers are preferred over natural fibers because of their resistance to decomposition, high strength, and improved bond properties. Different types of artificial fibers have been added to concrete; some of the most commonly used are steel, glass, and polypropylene fibers. Research using fibers like polypropylene fiber, steel fiber, glass fiber, carbon fiber, polyvinyl alcohol fiber, etc., has reported successful results [18,19,20,21,22,23].

Steel fibers in concrete are known to improve its mechanical properties, flexural post-cracking behavior, bond strength, fracture properties, and long-term durability [24,25,26,27,28]. Using steel fibers in SSC reduces early drying shrinkage and improves the durability of the concrete [29,30]. However, steel fibers have higher stiffness than cement paste, which may initiate micro-cracks during hydration [30]. Further, a significant issue that poses a risk to the long-term performance of steel-fiber-reinforced concrete is the fibers’ tendency to rust, especially in an aggressive environment [31]. A previous study has reported a reduction in corrosion problems by using galvanized steel fibers [32]. It has been reported that steel fibers, when added to concrete, can increase its porosity, allowing aggressive ions to enter deep into the concrete [33], which can further aggravate the corrosion problem. With the increase in steel fiber corrosion, a reduction in its ability to control cracks and a rapid increase in crack width has been reported [34]. Many research studies have been carried out on the durability of steel-fiber-reinforced concrete, and it is concluded that steel fiber bridging cracks wider than 0.5 mm have a significant risk of corrosion [31,35,36]. Thus, severe corrosion of the steel fibers can significantly reduce the fibers’ cross-sectional area and the composites’ residual tensile strength [37].

Another common fiber added to concrete is glass fibers, and it is reported that they bridge micro-cracks in the concrete matrix, preventing stresses from spreading at the crack tip and realizing the failure mode of “cracking without fracture,” thus improving the mechanical performance of concrete [38,39,40]. It has been reported that increasing the glass fiber content improves the crack shrinkage effect and crack width at the optimum value of 1% v/f of the fiber content [41]. Adding glass fibers into SCC improves its mechanical properties and permeability characteristics by refinement of the pore structure of the matrix [42]. However, one of the issues with glass fibers is their brittle nature, thus breaking during the concrete mixing; therefore, extreme care is required during the mixing process. The other serious issue is their reactivity in alkaline environments such as that of concrete. The corrosion process of glass fibers in alkaline solutions is mainly determined by breaking the glass-forming -Si-O-Si- bonds, leading to a completely destroyed network [43].

Thus, polypropylene fibers have been used in concrete due to their ease of application, low density, non-breakability, corrosion resistance, and other benefits. The addition of polypropylene fibers to concrete improves its mechanical and durability properties and provides better resistance to thermal stresses and increased energy absorption [44,45]. Further, the fatigue life of polypropylene-fiber-reinforced concrete (PFRC) is significantly improved compared with that of plain concrete [46,47]. The use of polypropylene fibers in SCC significantly affects the resulting concrete’s mechanical properties and crack propagation [48,49,50]. However, traditionally available polypropylene fibers are tiny, have smooth surfaces, and are very soft. Because of their smooth surface, their bonding with the surrounding concrete may be affected, and their soft nature may lead to a balling effect during the mixing of concrete, giving rise to uneven fiber distribution and affecting concrete performance. To cater to these problems, macro-polypropylene fibers have been used. Using 0–1% of macro PP fiber in normal-strength concrete, a maximum increase in compressive strength of up to 17.5% has been noted at the fiber content of 0.5% v/f [51]. Owing to twisted-bundle monofilament macro PP fiber’s bridging effect, its addition to concrete significantly improves its resistance against freezing and thawing [52]; at the same time, another study reported an improvement of 32.78% in the tensile strength of normal-strength concrete by using 38 mm macro PP fibers at 0.6% v/f [53]. Research utilizing waste macro PP fibers of 30 mm length and a fiber content of 0–1.25% v/f has reported significant improvements in resistance to sulfate and acid attacks by 30.39% and 28.47% [54].

The macro PP fibers mostly used in previous studies have smooth surfaces; however, recently, a new type of macro-polypropylene fiber has been developed by gluing microfibers together and providing undulations on the surfaces of the fibers, which may result in better bonding because of mechanical anchorage, similar to steel fibers. As an alternative to steel and glass fibers, which are degraded over time because of extreme exposure and affect the long-term durability of concrete, macro-polypropylene fibers may be the preferred option. Thus, these fibers are added to self-compacting concrete to investigate its durability under severe exposure, including freezing and thawing cycles, sulfate attack, and acid attack.

## 2. Research Aims, Scope, and Novelty

Commonly used artificial fibers include glass, steel, and polypropylene fibers. Glass fibers have some associated problems. They are reactive in alkaline environments, like in concrete, and further, they are brittle, so breaking occurs during the concrete mixing process. On the other hand, steel fibers are known for corrosion problems in specific environmental conditions.

Polypropylene fibers may be a solution to these issues. However, traditional polypropylene fibers are very soft, and the balling effect occurs during the concrete mixing process, leading to uneven fiber distribution. Further, they have smooth surfaces, which may lead to weaker bonds in concrete because of no mechanical anchorage. To solve these problems, a new type of polypropylene fiber is developed by gluing micro-polypropylene fibers together and providing undulations on the surface for mechanical anchorage to form macro-polypropylene fibers. The resulting fibers have high strength, low density, better bonding properties, are non-corrosive, and are unbreakable. To the authors’ knowledge, because it is relatively new, limited studies have been conducted on this type of fiber addition on the durability properties of self-compacting concrete (SCC). Thus, this study investigates the effect of different amounts of macro-polypropylene fiber addition into different grades of concrete on fresh concrete properties, including workability, density, and air content. Additionally, the durability of SCC is investigated under exposure to freezing and thawing cycles, sulfate attack, and acid attack.

## 3. Materials

Materials for concrete were collected from the local market. Ordinary Portland Cement CEM-I 32.5N having a specific surface of 235 m^2^/kg was used, conforming to ASTM C150 [55] standard. The chemical composition of the cement used is presented in Table 1.

Normal-weight crushed coarse aggregates (CAs) and river sand as fine aggregates were used. Tests were conducted per ASTM [56,57,58] standards for the basic properties of the materials used for finalizing the concrete mix design and these are presented in Table 2.

**Figure 1:**
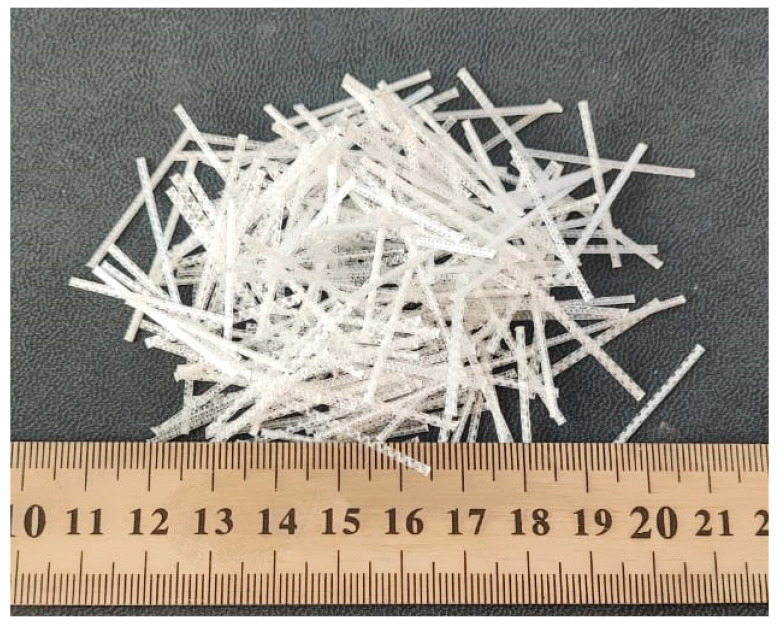
Macro-polypropylene fibers.

SIKA, Islamabad, Pakistan provided macro-polypropylene fibers (MPFs) and polycarboxylate-based superplasticizers (SPs) with a trade name of “SIKA Viscocrete 3110”. The properties of the fibers, as provided by the supplier, are presented in Table 3, and the fibers are shown in Figure 1.

## 4. Methodology

**Figure 2:**
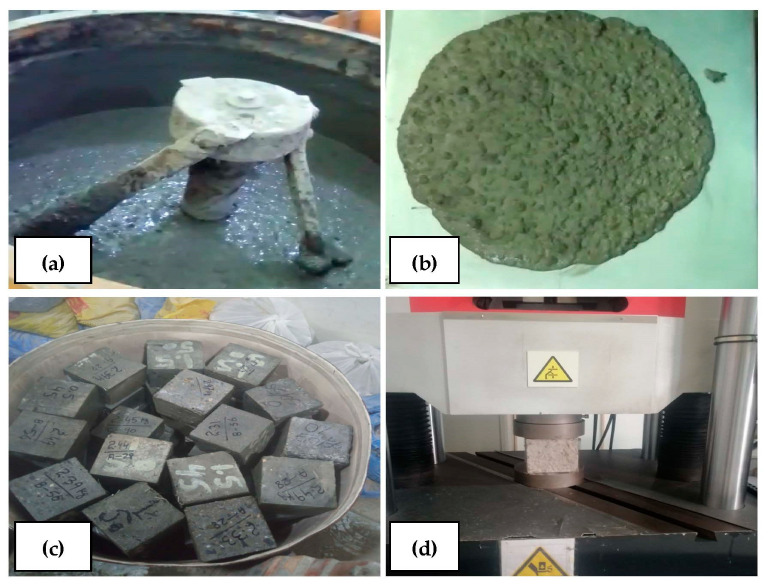
(**a**) Mix preparation using a pan mixer; (**b**) slump flow test; (**c**) samples curing in water tank; (**d**) experimental test setup using a universal testing machine.

Essential concrete mixes for three grades of self-compacting concrete with targeted strengths of 25 MPa, 30 MPa, and 35 MPa were finalized using EFNARC guidelines [59] and performing trial mixes. Fibers were added to each concrete grade in 0.5%, 1%, and 1.5% v/f to compare their performance with the base mix without fibers. The workability of the concrete was studied by conducting slump flow tests as per ASTM C1611/C1611M [60] with a target minimum slump flow of 600 mm. Air content and fresh concrete densities were noted using ASTM C138/C138M [61]. A total of 432 concrete cubes with dimensions 100 mm × 100 mm × 100 mm were cast and tested at the concrete ages of 28 days, 56 days, and 92 days. The samples were demolded after 24 h of casting and then transferred into water tanks for moist curing per ASTM C192/C192M [62]. All the samples were cured in regular tap water for the first 14 days, assuming an early exposure to extreme conditions on-site, and then transferred to acid and sulfate solutions and for freezing and thawing cycles, leaving behind the reference concrete samples. A 10% hydrochloric acid solution and a 10% sodium sulfate solution were used. The freezing and thawing cycles were kept at 12 h of freezing in the refrigerator and then 12 h of thawing by taking the sample out. All the samples were tested at 28 days, 56 days, and 92 days for compressive strength. The results were then analyzed and reported. Figure 2 shows the mix preparation, slump flow, curing, and experimental setup. The experimental test setup included a universal testing machine, “Ibertest”, which had a capacity of 2000 KN applying continuous load. A stress-controlled setup was used, applying continuously increasing stress at the rate of 0.5 MPa/s as prescribed in the UTM manual. The setup also included a monitor showing the loading process and the stress levels.

## 5. Concrete Mix Design

EFNARC guidelines [59] for self-compacting concrete were used, trial mixes were conducted to finalize the concrete mix design for different grades of concrete, and then macro-polypropylene fibers were added to them in different proportions. Various researchers have used varying amounts of fiber percentages in concrete and mortar. Face-mask fibers have been utilized in cement mortar at 0.10, 0.15, 0.20, and 0.25%, exhibiting the best performance at 0.15% fiber content [63]. Similarly, another study concluded that the average best dosage of polypropylene fiber ranges from 1 to 2% depending on the diameter, length, and concrete mix design [64]. The mix proportions for all the concretes are summarized in Table 4. The nomenclature used for concrete, e.g., C25, refers to “C” for concrete, then 25 for a concrete grade with 25 MPa compressive strength.

## 6. Results and Discussion

### 6.1. Properties of Fresh Mix

The fresh concrete properties, including workability using the slump flow test, fresh concrete density, and air content, are summarized in Table 5.

**Figure 3:**
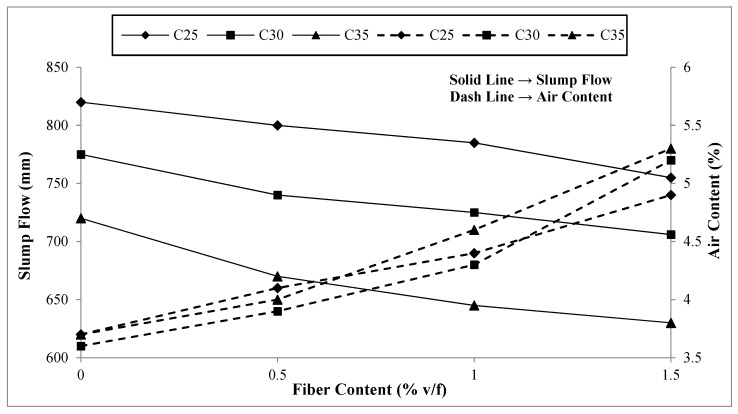
Slump flow and air content.

As can be observed from the test results, there is no significant difference in the fresh concrete density, and the variation is within 5%. However, there is some variation in the slump flow and significant variation in the air content of the concrete. The test results of the slump flow and air content are presented in Figure 3.

The slump flow reduces as the macro-polypropylene fibers are incorporated into the concrete, increasing its content. It reduces by 8%, 9%, and 12.5% for C25, C30, and C35 when the fiber content increases from 0% to 1.5%. However, all the concrete slump flows are still above the target range of 600 mm. It can be observed from the slump flow values that the variation is not as significant as that reported by the previous study on steel fiber addition to SCC [4]. The reason may be a lower hindrance to concrete flow because of their smooth surface compared to steel fibers.

Regarding the test results for the air content, there are significant variations with the addition of macro-polypropylene fibers into concrete. The air content increased by 32%, 45%, and 43% for C25, C30, and C35 when 1.5% v/f of fibers were added compared to their respective reference concretes with zero fiber content. This increase may be attributed to the hindrance provided by the fibers to the compaction of the concrete and air bubbles entrapped around the undulations provided on the surface of the fibers for improving their bonding characteristics. Although there is a significant increase in the air content with fiber addition, there is no major variation in the density of the resulting concrete. The reason is that the air content makes up a tiny proportion of the concrete and, even if increased drastically, affects the overall density by merely a few percent. Previous studies have reported similar results with a considerable increase in air content and an insignificant change in the density of the concrete with fiber addition [4,5,6,7,65].

### 6.2. Hardened Concrete Test Results

Tests were conducted on samples subjected to normal curing and others exposed to various environmental conditions like freezing and thawing and acid and sulfate attacks. For this purpose, a rigorous experimental program was planned, and 432 cube samples were tested in total. The reason for testing cube samples was to have equal exposure from all sides of the samples. The universal testing machine was calibrated before starting the experimental program to avoid any potential error or deviation in results. Further extreme care was exercised during the tests in applying loads and recording the results. The presented test results are the average of three samples for each test, and the data used were well within the limit to qualify for the use of average value calculation with no significant variance. The test results for freezing and thawing, sulfate, and acid attacks are presented and discussed in the following sections. The compressive strengths of the control samples not exposed to any type of extreme conditions are presented in Table 6.

#### 6.2.1. Freezing and Thawing

**Figure 4:**
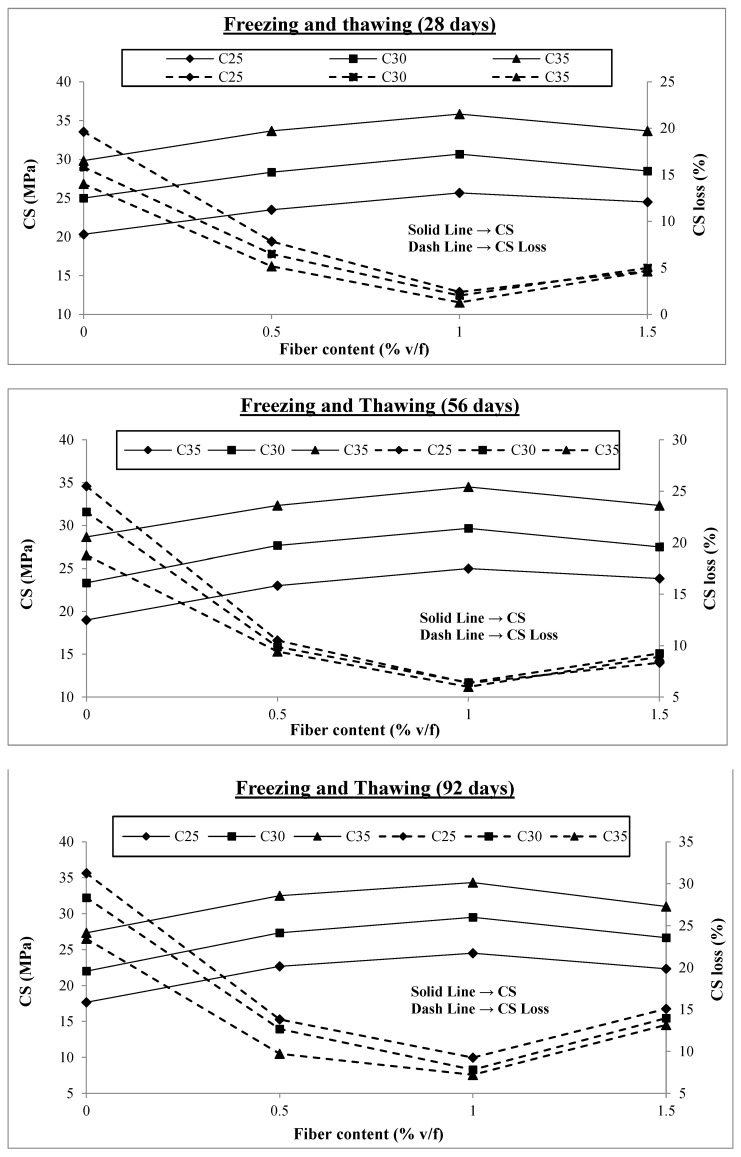
Compressive strengths under freezing and thawing.

Figure 4 shows the test findings for samples with varying fiber content and those without fibers subjected to freezing and thawing cycles. All samples were moist-cured for 14 days and then exposed to freezing and thawing cycles. The freezing period was for 12 h at −15 °C followed by 12 h of thawing in the water tank at room temperature until testing day, i.e., 28, 56, and 92 days. In total, 108 samples were tested and the results were examined. The tests’ findings have various outcomes, which are discussed.

Figure 4 shows that concrete’s compressive strength significantly decreases when subjected to freezing and thawing cycles. For C25, C30, and C35, the maximum compressive strength losses are 31.1%, 28.3%, and 23.4% at the concrete age of 92 days, respectively. However, these losses were reduced considerably by incorporating macro PP fibers into the concrete. The maximum compressive strength loss reduction is 70%, 72%, and 69% for C25, C30, and C35, respectively, at 92 days when adding 1% v/f of macro PP fibers into them. A study reported improving the freezing and thawing resistance by using twisted-bundle monofilament macro PP fibers in concrete [52]. Another study reported a reduction in the compressive strength loss of SCC subjected to 50 freeze–thaw cycles by 27.3% with the addition of 0.20% polypropylene fiber [62]. All 1% fiber content levels resulted in the most significant decreases in compressive strength loss. Beyond this value, the strength loss starts to increase again. During this exposure, water seeps into the pores of the concrete, and when it freezes, it exerts hydraulic pressure inside the concrete, leading to the development of micro-cracks. After thawing, more water seeps into the micro-cracks, increasing the hydraulic pressure inside the concrete, widening the cracks, and ultimately causing the disintegration of the structure. If the concrete is strong enough to withstand the tensile stresses brought on by internal hydraulic pressures, no cracks will occur, obstructing further water infiltration. The well-known fact about the fibers’ bridging effect, which slows the formation of cracks and prevents their spread, may account for the greater resilience of concrete to freezing and thawing with fiber inclusion, resulting in enhanced performance in the face of such intense exposure [66].

**Figure 5:**
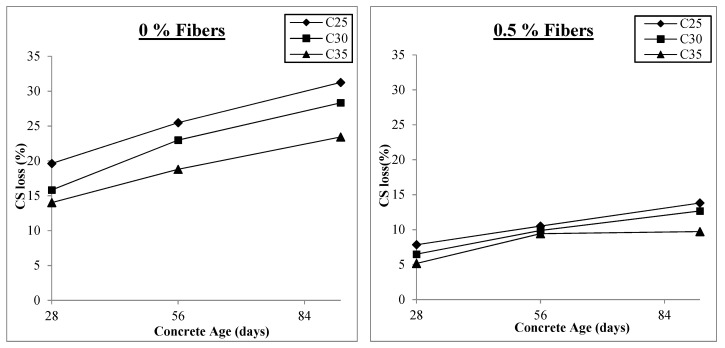

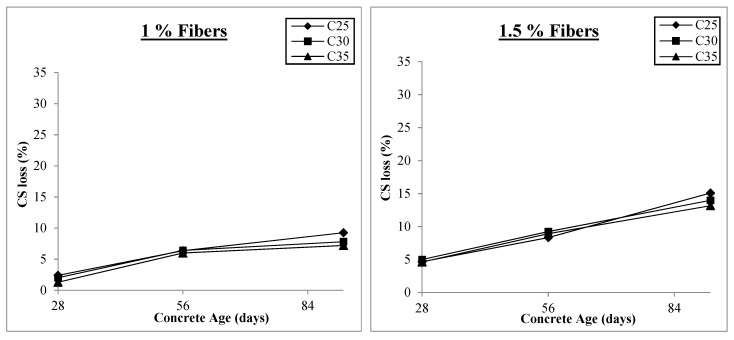
Compressive strength loss under freezing and thawing.

The other significant discovery from the test findings is that, as shown in Figure 4, strength losses are smaller in concrete with higher compressive strength than in concrete with lower compressive strength, which may be attributed to the better bonding of fibers in higher-strength concrete. Therefore, C35 performs better than C25 because increased compressive strength also means increased tensile strength of concrete, which increases the concrete’s resistance and results in better performance. Furthermore, it is clear from Figure 4 that the difference in compressive strength losses is greater with 0% fibers in different concrete classes and gets smaller as fiber content increases. This is because, while no fibers are present, only the tensile strength of plain concrete can withstand hydraulic pressure. However, as the fiber content rises, the fibers’ activity provides resistance, negating the impact of the concrete’s tensile strength. The results for compressive strength losses for fiber concentrations of 0%, 0.5%, 1%, and 1.5% are shown in Figure 5. In order to show the change in compressive strength loss with the addition and growth in fiber content, the y-axis range of all the graphs is held at the same level.

As previously mentioned, the loss of compressive strength is initially at its highest level and begins to decrease as macro-polypropylene fibers are added to the concrete, reaching its lowest values at 1% v/f of fibers before beginning to increase. Figure 5 shows that for all concrete classes at 0% fiber content, the compressive strength loss varies between 14% and 31%. This range narrows to 5% to 14% at 0.5% fiber content and minimal values of 1% to 9% at 1% v/f of fibers in concretes. The compressive stress loss range increases to 4% and 15% when the fiber concentration is further raised to 1.5% v/f, similar to that of 0.5% fibers. Therefore, fiber addition above 1% harms concrete’s ability to withstand freezing and thawing. The increase in air content within the concrete could cause this adverse effect. Concrete becomes more porous as its air content rises, facilitating water infiltration and crack development when subjected to freezing and thawing. Although this effect still occurs when fewer fibers are present, the increase in air content happens gradually, and the fibers mitigate its adverse effects. However, when the fiber content is raised from 1% to 1.5%, there is a sharp increase in the air content values, which lessens the effect of fibers. Additionally, when the amount of fiber is above a certain optimum level and the amount of air is increased, more voids are trapped around the surface of the fibers and there is relatively less fiber contact with the concrete, resulting in lower bond strength. Thus, 1% of macro-polypropylene fibers may be considered the optimum value for action against freezing and thawing.

#### 6.2.2. Sulfate Attack

**Figure 6:**
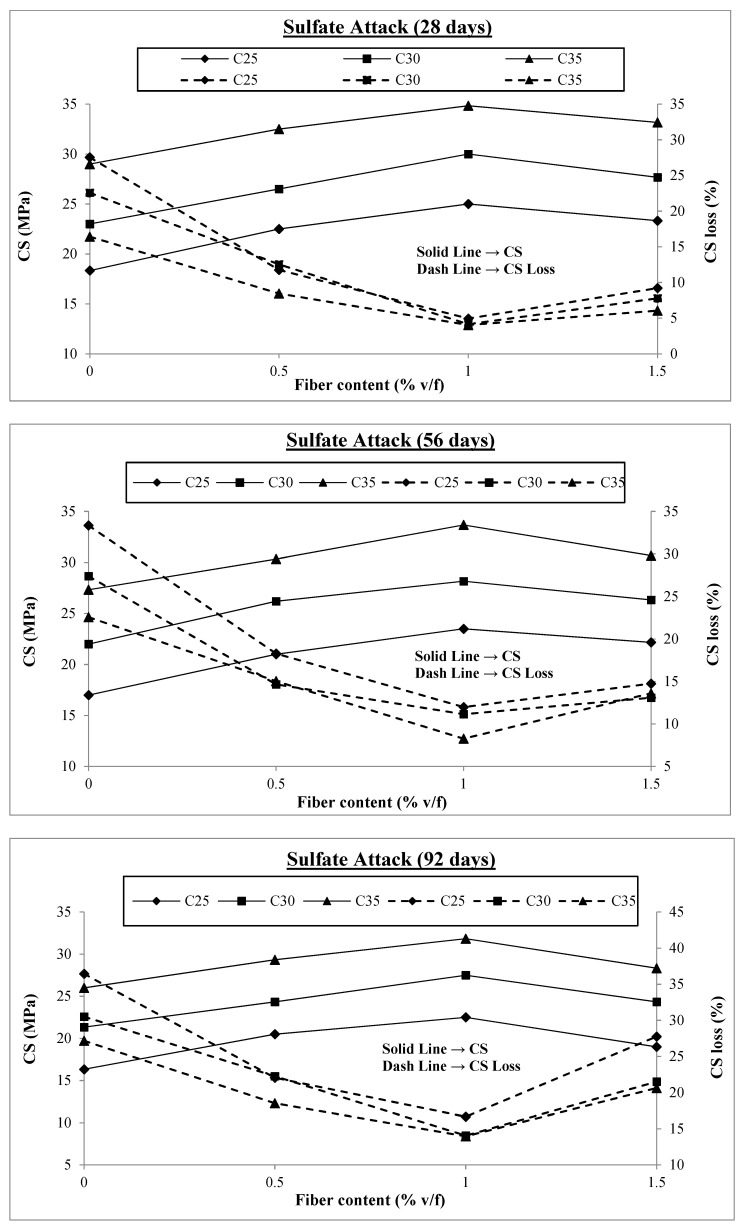
Compressive strengths under sulfate attack.

The test results of the samples exposed to 10% sodium sulfate solution having no fibers and different v/f of fibers are presented in Figure 6. All the samples were initially subjected to normal curing in the first 14 days and then exposed to sulfate attack. In total, 108 samples were tested under this condition. The outcomes of sulfate attack tests at concrete ages of 28 days, 56 days, and 92 days are discussed in the following section.

It may be observed from Figure 6 that there is a significant loss in the compressive strength of concrete when exposed to sulfate attack. The maximum loss in compressive strengths at the concrete ages of 28 days, 56 days, and 92 days are 27.5%, 33.3%, and 36.4%; those were for C25 concrete containing no fibers. This means that almost one-third of the concrete strength is lost. However, with the addition of macro-polypropylene fibers to the concrete, this negative impact of sulfate attack is significantly reduced. The maximum reductions in compressive strength loss are all with 1% fiber content and at the concrete age of 92 days. These reductions are 54%, 54%, and 48% for C25, C30, and C35, respectively. Beyond this value, the strength loss starts to increase again. When exposed to 5% sodium sulfate solution, there is a reduction in compressive strength loss from 20% to 11% with the addition of 0.3% of PP fibers by weight of the cement into SCC [67]. Similarly, another study reported that PP fibers in SCC incorporated at 0.10% along with 20% of granite powder exhibit 25.4% improved strength at 90 days of exposure to sulfate solution [29]. Another study reported that using waste macro PP fibers of 30 mm length by 1% v/f improves the compressive strength loss reduction by 30.39% against sulfate attack [54]. In the case of freezing and thawing cycles, the strength loss at 1.5% fiber content was similar to that for concrete with 0.5% fiber content; however, in the case of sulfate attack, the compressive strength loss with 1.5% fiber content at 92 days is even more than that for concrete containing 0.5% fibers. Other researchers reported similar trends of SCC incorporated with polypropylene fibers [27,68]. In this kind of exposure, the sulfate solution seeps into the concrete and reacts with the hydrated cement paste to form calcium sulfoaluminate or ettringite. The volume of ettringite is more than that of the original compounds reacting to form it, so it exerts pressure within the concrete similar to the hydraulic pressure in freezing and thawing, leading to crack formation. These internal stresses are resisted by the concrete’s tensile strength and the additional strength provided by the fibers’ bridging effect.

**Figure 7:**
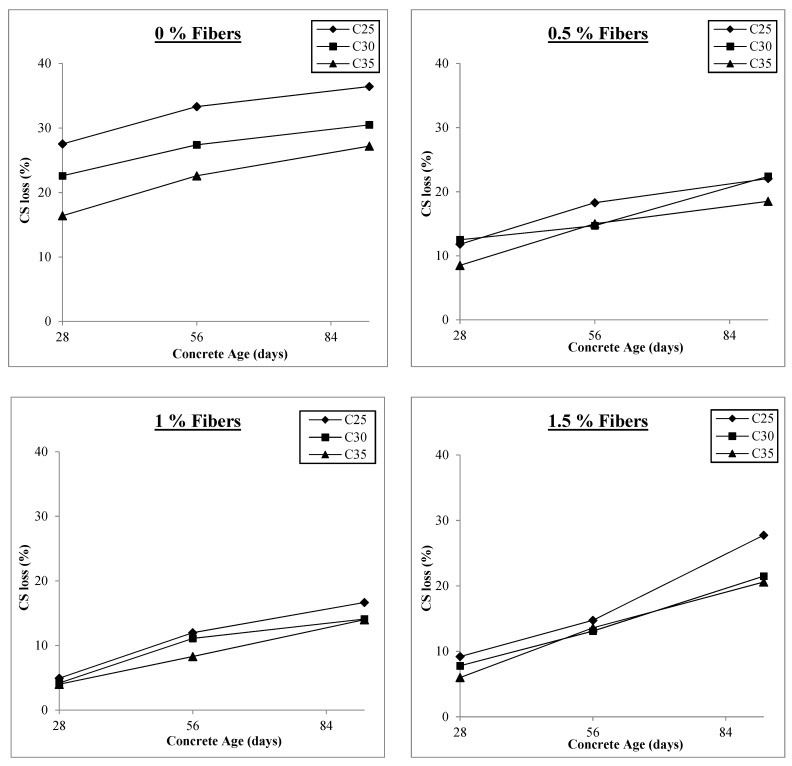
Compressive strength loss under sulfate attack.

Another important discovery from the test results is that similar to freezing and thawing, strength losses are smaller in concrete with higher compressive strength than in concrete with lower compressive strength at the same fiber content. Due to the resistance offered by the fibers and the fact that higher compressive strength also translates to improved tensile strength of concrete, C35 performs better than C25. Furthermore, it is clear from Figure 6 that the difference in compressive strength losses is higher with 0% fibers and decreases with fiber content increasing to 1%, demonstrating the best results. This is because, while there are no fibers in the concrete, only its tensile strength can withstand hydraulic pressure; however, as the fiber content rises, the fibers’ bridging activity may now give extra resistance, negating the impact of the concrete’s tensile strength. The results for compressive strength losses are shown in Figure 7 for fiber contents of 0%, 0.5%, 1%, and 1.5%.

As already discussed, the compressive strength loss is initially at a maximum level and starts reducing with the addition of macro-polypropylene fibers to the concrete, achieving minimum values at 1% v/f of fibers and then increasing. Figure 7 shows that the compressive strength loss varies between 16% and 36% for all the concrete classes at 0% fiber content. At 0.5% fiber content, this range reduces to 2% to 22%, reducing to minimum values of 4% to 17% at 1% v/f of fibers in concretes. When the fiber content is further increased to 1.5% v/f, the compressive strength loss range again increases to between 6% and 28%, which is even higher than those with 0.5% fiber content. Thus, the effect of fiber addition beyond 1% is detrimental to concrete resistance against sulfate attack. Looking at the air content values, when the fiber content is increased from 1% to 1.5%, there is a sudden jump in air content values, reducing the effect of fiber action. Additionally, increased fiber content beyond a specific optimum value and increased air content result in more voids entrapped around the fiber’s surface and comparatively lesser contact of fibers with concrete, leading to lower bond strength. Further, the sulfate attack gets more severe with the increased porosity of concrete. Because of this increased permeability and immersion in sulfate solution, the calcium hydroxide in concrete is leached out, making the concrete more permeable, making it easier for the sulfate solution to seep through as time passes, and causing disintegration of the concrete. This may be the reason for more strength loss for concretes containing 1.5% fibers at a concrete age of 92 days compared to those with fewer fibers.

#### 6.2.3. Acid Attack

**Figure 8:**
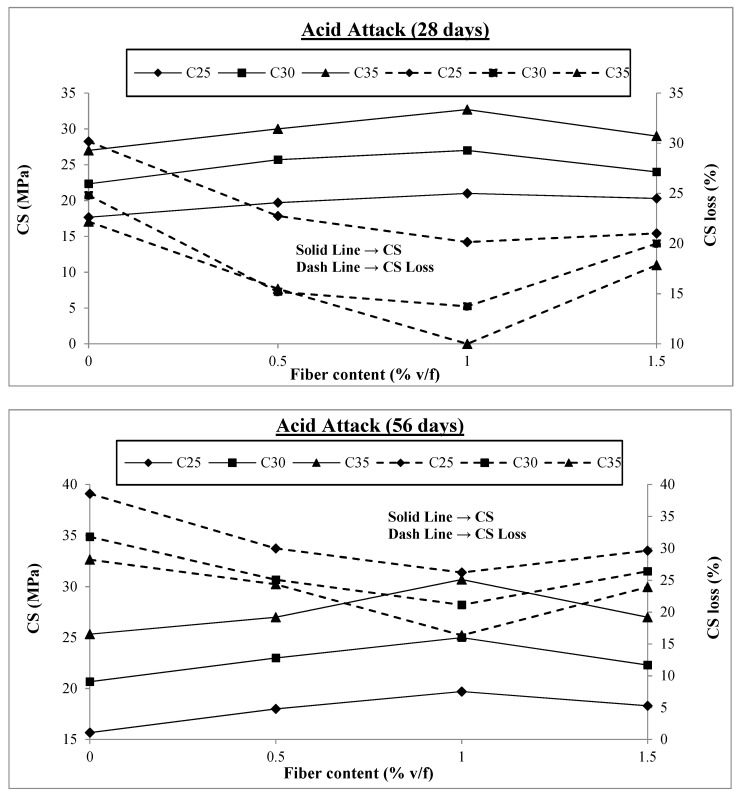

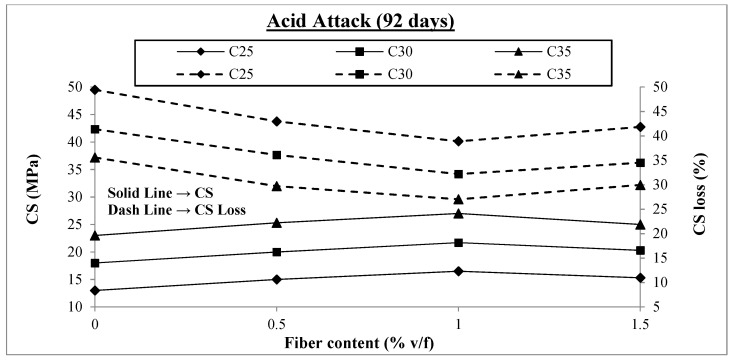
Compressive strengths under acid attack.

The test results of the samples exposed to acid attack having no fibers and different v/f of fibers are presented in Figure 8. Similar to exposure to freezing and thawing and sulfate solution, all the samples were subjected to normal curing in the first 14 days and then exposed to a 10% hydrochloric solution until testing. The outcomes from the test results are discussed.

It can be observed from Figure 8 that there is a significant loss in the compressive strength of concrete when exposed to acid attack, more than that of freezing and thawing and sulfate attack. The maximum loss in compressive strength at the concrete age of 92 days is 49.4%, which is for C25 concrete containing no fibers. This means that half of the concrete strength is lost at the concrete age of 92 days. However, with the addition of macro-polypropylene fibers to concrete, this negative impact of acid attack is reduced but not to the same extent as in the case of freezing and thawing and sulfate attack. The maximum reductions in compressive strength loss are all with 1% fiber content, and at a concrete age of 92 days, these reductions are 21%, 22%, and 24% for C25, C30, and C35, respectively. Beyond this value, the strength loss starts to increase again. A study investigated the effect of NaCl, MgSO_4,_ and H_2_SO_4_ solutions on the compressive strength loss of self-compacting fiber-reinforced concrete when immersed for 120 days. The NaCl solution did not affect the compressive strength; however, H_2_SO_4_ and MgSO_4_ solutions reduced compressive strength by 12.9–40.6% and 12.1–15.8%, respectively [69]. Another study reported that using waste macro PP fibers of 30 mm length by 1% v/f improves the compressive strength loss reduction by 28.47% against acid attack [54]. Similarly, another study reported that PP fibers in SCC incorporated at 0.10% exhibit 16.5% strength loss reduction at 90 days of immersion in 3% dilute sulfuric acid [67]. In the current study, similar to the test results for freezing and thawing, the compressive strength loss with 1.5% fiber content at 92 days is similar to that for concrete containing 0.5% fibers. Other studies also support these research findings [18,29]. Concrete disintegration in an acid attack differs from that of freezing and thawing and sulfate attack, where the main disintegration is caused by internal stresses caused by pressure inside the concrete. However, in the case of an acid attack, the cement paste is directly attacked by the acid solution, disintegrating the hydrated cement paste, which is dissolved into the acid solution and thus removed, leaving behind a soft and weak mass. The impact of fiber addition to concrete is not significant because fibers only play a secondary role in maintaining the integrity of concrete by binding it together and cannot resist the acid attack directly, unlike in the cases of freezing and thawing and sulfate attack where the bridging effect of fibers plays a vital role in resisting the internal tensile stresses and reducing the impact of severe exposure.

**Figure 9:**
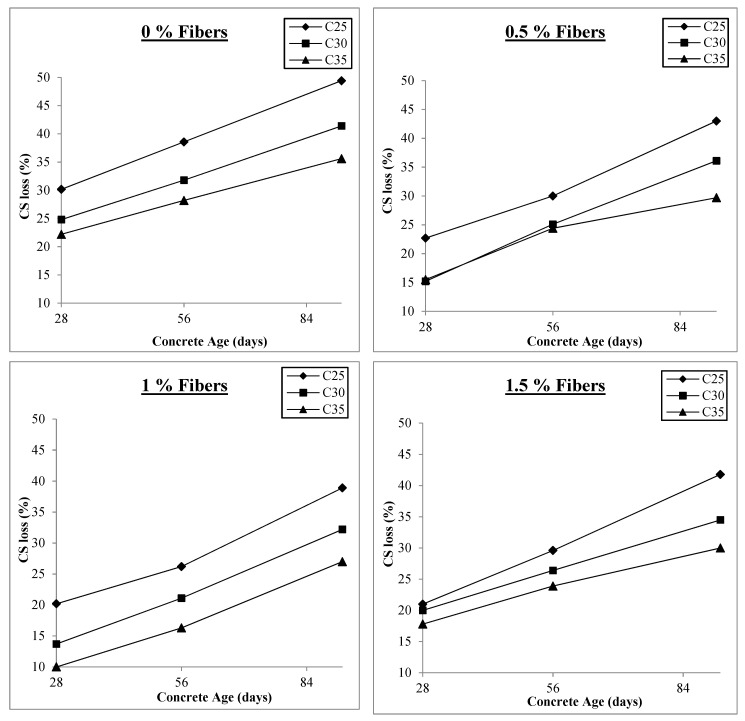
Compressive strength loss under acid attack.

Like freezing and thawing and sulfate attack, the strength losses are less in concrete with higher compressive strength than in concrete with lower compressive strength at the same fiber content. Thus, C35 performs better than C25 because its higher compressive strength results from more cement paste and better quality, which takes longer to be disintegrated by acid attack. Figure 9 presents the results for compressive strength losses at 0%, 0.5%, 1%, and 1.5% fiber contents.

As already discussed, the compressive strength loss is initially at a maximum level and then starts reducing with the addition of macro-polypropylene fibers to the concrete, achieving minimum values at 1% v/f of fibers and then increasing. Figure 9 shows that the compressive strength loss varies between 22% and 49% for all the concrete classes at 0% fiber content. At 0.5% fiber content, this range reduces to 15% to 43%, reducing to minimum values of 10% to 39% at 1% v/f of fibers in concretes. When the fiber content is further increased to 1.5% v/f, the compressive strength loss range rises again to 18% and 42%, similar to that with 0.5% fiber content. Thus, although fiber addition improves the resistance of concrete against acid attack, it is not significant, and fiber addition beyond 1% is detrimental to concrete’s resistance against acid attack. There is a sharp increase in air content when the fiber content is increased from 1% to 1.5%, lessening the effect of fiber action and lowering the contact between the fibers and the surrounding concrete, resulting in decreased bond strength. In addition, increased permeability makes the acid attack more severe since it is easier to penetrate deep into the concrete, which is likely the source of more significant strength loss for concretes containing 1.5% fibers at a concrete age of 92 days compared to those containing 1% fibers.

## 7. Conclusions

This research study investigated the effect of adding macro-polypropylene fibers to different strength concretes on their fresh and durable properties. The following conclusions may be drawn based on this study:

1. There is a reduction in the slump flow of concrete when macro PP fibers are added to concrete, and its content is further increased because fibers hinder concrete flow. However, this reduction is not as significant as that of steel fibers, which may be because the comparatively smoother surface of PP fibers reduces surface friction during concrete flow.
2. There is a significant increase in the air content of concrete when macro-polypropylene fibers are added to it. This significant increase may be attributed to the hindrance fibers provide to concrete compaction and, most notably, the undulations on the surface of fibers facilitating the entrapment of air around these undulations.
3. The resistance of concrete containing macro-polypropylene fibers to freezing and thawing cycles is significantly improved up to a fiber content of 1% and then starts to decrease. The resistance in this study improved by up to 72% at 92 days. This significant increase may be attributed to the improved bridging effect of fibers because of the enhanced mechanical anchorage provided by the fibers’ surface undulations. However, beyond the optimal fiber content, its performance starts to degrade, which is 1% in this study.
4. The addition of macro-polypropylene fibers to concrete significantly increases its resistance against sulfate attacks due to their bridging effect, like in freezing and thawing. The maximum resistance is up to 54% at a 1% fiber content, but this resistance reduces significantly after the fiber content is increased beyond the optimal level because of the increase in the porosity of the concrete facilitating the sulfate ingress.
5. The addition of macro-polypropylene fibers to concrete improves its resistance against acid attack, however, to a lesser extent compared to freezing and thawing cycles and sulfate attack. The maximum resistance of up to 24% is achieved with a 1% fiber content and starts to lower with a further increase in fiber content because the increase in the porosity of the concrete makes the attack more severe. The lower resistance in the case of acid attack may be because, in this case, the cement paste is directly attacked by acid, disintegrating it, and thus, the fiber bridging effect is of lower importance.
6. Higher-strength concrete possesses more resistance to severe exposures like freezing and thawing, sulfate, and acid attacks because of better concrete quality and improved bonding of fibers in concrete, boosting its performance.

As significant improvements have been noted in this study in the performance of normal-strength concrete when exposed to severe environmental conditions, there is a need to investigate these properties further using high-strength and ultra-high-strength concrete for specialized applications. Further, the aspect ratio of fibers is known to alter the performance of fibers in concrete; macro PP fibers with different aspect ratios may be used to study their performance.

## Figures and Tables

**Table 1:** Chemical composition of cement.

Constituents	% Age by Weight
Lime (CaO)	60.24
Silica (SiO_2_)	19.57
Alumina (Al_2_O_3_)	5.95
Iron Oxide (Fe_2_O_3_)	3.96
Magnesia (MgO)	3.71
Sulphur Trioxide (SO_3_)	2.81
Sodium Oxide (Na_2_O)	0.93
Loss on ignition	1.92
Insoluble residue	0.91
C3S	42.8
C2S	23.9
C3A	9.1
C4AF	12

**Table 2:** Material properties.

Material	Size (mm)	Water Absorption (%)	Specific Gravity
Fine Aggregate	0–2	1.3	2.60
Coarse Aggregate	2–12.5	1.1	2.62
Fly Ash	-	-	2.08
Superplasticizer	-	-	1.08

**Table 3:** Properties of macro-polypropylene fibers.

Sr. No.	Description	Property
1	Color	Natural white
2	Length	30 mm
3	Width	1.24 mm
4	Density	910 kg/m^3^
5	Melting point	160 °C
6	Ignition point	350 °C
7	Thermal and electrical conductivity	Low
8	Absorption	Nil
9	Tensile strength	465 N/mm^2^
10	Modulus of elasticity	7.5 kN/mm^2^

**Table 4:** Concrete mix composition.

Concrete Type	Quantities (kg/m^3^)						
	MPF	Cement	Fly Ash	Super Plasticizer	Coarse Aggregate	Fine Aggregate	Water
C25	0	400	100	4	915	650	170
	4.55	400	100	4	915	650	170
	9.1	400	100	4	915	650	170
	13.65	400	100	4	915	650	170
C30	0	400	100	4.75	915	650	160
	4.55	400	100	4.75	915	650	160
	9.1	400	100	4.75	915	650	160
	13.65	400	100	4.75	915	650	160
C35	0	400	100	5.75	915	650	150
	4.55	400	100	5.75	915	650	150
	9.1	400	100	5.75	915	650	150
	13.65	400	100	5.75	915	650	150

**Table 5:** Fresh concrete properties.

Concrete	Fiber Content (% v/f)	Slump Flow (mm)	J-Ring (mm)	L-Box Value	V-Funnel Time (Sec)	Density (kg/m^3^)	Air Content (%)
C25	0	820	800	0.98	6	2410	3.7
	0.5	800	760	0.89	8	2395	4.1
	1	785	730	0.85	11	2385	4.4
	1.5	755	680	0.82	15	2350	4.9
C30	0	775	750	0.96	7	2415	3.6
	0.5	740	695	0.87	10	2390	3.9
	1	725	665	0.84	12	2380	4.3
	1.5	705	630	0.80	17	2365	5.2
C35	0	720	690	0.92	8	2425	3.7
	0.5	670	625	0.86	11	2395	4
	1	645	600	0.81	14	2370	4.6
	1.5	630	540	0.76	20	2350	5.3

**Table 6:** Compressive strengths of control samples.

Concrete	Fiber Content (% v/f)	Compressive Strength (MPa)		
		28 Days	56 Days	92 Days
C25	0	24.3	26	26.9
	0.5	24.5	26.2	27.6
	1	25.3	27.5	28.1
	1.5	24.7	26.5	27.4
C30	0	28.7	30.9	32.3
	0.5	29.3	31.2	32.6
	1	30.3	32.4	33.2
	1.5	29	30.8	32.4
C35	0	33.7	35.8	36.9
	0.5	34.5	36.3	37.1
	1	35.3	37.2	38.2
	1.5	34.3	36	37.2

## Data Availability

Data are contained within the article.
